# Inhibition of MLKL-dependent necroptosis via downregulating interleukin-1R1 contributes to neuroprotection of hypoxic preconditioning in transient global cerebral ischemic rats

**DOI:** 10.1186/s12974-021-02141-y

**Published:** 2021-04-20

**Authors:** Lixuan Zhan, Xiaomei Lu, Wensheng Xu, Weiwen Sun, En Xu

**Affiliations:** Institute of Neurosciences and Department of Neurology of The Second Affiliated Hospital of Guangzhou Medical University and Key Laboratory of Neurogenetics and Channelopathies of Guangdong Province and the Ministry of Education of China, 250 Changgang Dong RD, Guangzhou, 510260 People’s Republic of China

**Keywords:** Necroptosis, MLKL, IL-1R1, Plasma membrane translocation, Calcium ion influx, Cerebral ischemia, Hypoxic preconditioning

## Abstract

**Background:**

Our previous study indicated that hypoxic preconditioning reduced receptor interacting protein (RIP) 3-mediated necroptotic neuronal death in hippocampal CA1 of adult rats after transient global cerebral ischemia (tGCI). Although mixed lineage kinase domain-like (MLKL) has emerged as a crucial molecule for necroptosis induction downstream of RIP3, how MLKL executes necroptosis is not yet well understood. In this study, we aim to elucidate the molecular mechanism underlying hypoxic preconditioning that inactivates MLKL-dependent neuronal necroptosis after tGCI.

**Methods:**

Transient global cerebral ischemia was induced by the four-vessel occlusion method. Twenty-four hours before ischemia, rats were exposed to systemic hypoxia with 8% O_2_ for 30 min. Western blotting was used to detect the expression of MLKL and interleukin-1 type 1 receptor (IL-1R1) in CA1. Immunoprecipitation was used to assess the interactions among IL-1R1, RIP3, and phosphorylated MLKL (p-MLKL). The concentration of intracellular free calcium ion (Ca^2+^) was measured using Fluo-4 AM. Silencing and overexpression studies were used to study the role of p-MLKL in tGCI-induced neuronal death.

**Results:**

Hypoxic preconditioning decreased the phosphorylation of MLKL both in neurons and microglia of CA1 after tGCI. The knockdown of MLKL with siRNA decreased the expression of p-MLKL and exerted neuroprotective effects after tGCI, whereas treatment with lentiviral delivery of MLKL showed opposite results. Mechanistically, hypoxic preconditioning or MLKL siRNA attenuated the RIP3-p-MLKL interaction, reduced the plasma membrane translocation of p-MLKL, and blocked Ca^2+^ influx after tGCI. Furthermore, hypoxic preconditioning downregulated the expression of IL-1R1 in CA1 after tGCI. Additionally, neutralizing IL-1R1 with its antagonist disrupted the interaction between IL-1R1 and the necrosome, attenuated the expression and the plasma membrane translocation of p-MLKL, thus alleviating neuronal death after tGCI.

**Conclusions:**

These data support that the inhibition of MLKL-dependent neuronal necroptosis through downregulating IL-1R1 contributes to neuroprotection of hypoxic preconditioning against tGCI.

**Supplementary Information:**

The online version contains supplementary material available at 10.1186/s12974-021-02141-y.

## Introduction

Ischemic stroke, often accompanied by high morbidity, mortality, and disability, is considered to be one of major causes of death and disability worldwide. Transient global cerebral ischemia (tGCI) is usually caused by acute heart failure, cardiac arrest, and shock etc. and leads to delayed neuronal damage in the hippocampal Cornu Ammonis 1 (CA1) subregion. Our previous studies demonstrated that both apoptosis and autophagy are involved in delayed neuronal death in CA1 after tGCI [1–3]. In recent years, accumulating evidence suggests that a novel type of cell death called programmed necrosis or necroptosis is associated with cerebral ischemic injury [4, 5]. We also reported that necroptosis contributes to hippocampal neuronal death after tGCI in adult rats. More specifically, our study further revealed that 30 min of hypoxic preconditioning applied 1 day before 10 min of tGCI-reduced necroptotic neuronal death in CA1 [6]. However, the precise mechanisms by which hypoxic preconditioning attenuates necroptotic neuronal death in CA1 region after tGCI remain poorly understood.

Necroptosis involves the formation of the necrosome, a necroptosis-inducing complex, containing the receptor interacting protein (RIP) 1, RIP3, and mixed lineage kinase domain-like (MLKL) [7, 8]. During the process of necroptosis, RIP1 and RIP3 form heterodimeric filamentous scaffold, which enables RIP3 to recruit and phosphorylate its substrate MLKL. RIP1 and RIP3 have been well documented in the regulation of necroptosis pathway. Our recent study demonstrated that the expression of RIP3 and the interaction of RIP1 and RIP3 increased in CA1 at the early stage of reperfusion after tGCI. Both hypoxic preconditioning and necrostatin-1 can decrease the expression of RIP3 and inhibit the interaction of RIP1 with RIP3 after tGCI, thereby attenuating necroptotic neuronal death in CA1 after tGCI [6]. Importantly, emerging evidence suggests MLKL to be the ultimate executor of necroptosis [9]. Therefore, it is worthwhile to explore whether hypoxic preconditioning alleviates hippocampal neuronal necroptosis after tGCI via a MLKL-dependent mechanism.

Although MLKL has been confirmed as a necroptotic effector downstream of RIP3, it is yet unclear how MLKL causes cell death. MLKL consists of an N-terminal four-helical bundle domain. This domain of MLKL is structurally similar to α-pore-forming toxins and is sufficient for oligomerization [10]. Mechanistically, the activation of MLKL requires phosphorylation of Ser345 and Ser347 in mouse MLKL. Activated MLKL translocates to the cell membrane and releases the four-helical bundle domain for oligomerization. Then, oligomeric MLKL interacts with phosphatidylinositol lipids within the plasma membrane, leading to membrane disruption and thus loss of ion homeostasis, cell swelling, and death [11–14]. It is intriguing that the membrane localization of MLKL promotes calcium ion (Ca^2+^) influx and ultimately induces necroptosis [11, 15]. However, a requirement for Ca^2+^ influx in necroptosis remains controversial. For some cell lines, Ca^2+^ influx after the activation of MLKL was necessary for the rupture of plasma membrane [11, 16, 17]. On the other hand, other ion such as Mg^2+^, K^+^, or Na^+^, not Ca^2+^, has been reported to be possibly involved in necroptotic cell death [14, 18, 19]. However, it remains to be established whether Ca^2+^ influx mediated by the activation of MLKL is involved in the hippocampal neuronal necroptosis after tGCI.

Currently, the specific mechanism for the regulation of MLKL phosphorylation and membrane localization has not been fully addressed. It is generally believed that necroptosis is influenced by the inflammatory milieu [20] and that interleukin-1 interacts with its receptor interleukin-1 type 1 receptor (IL-1R1) to directly induce an inflammatory response. Recent reports have demonstrated that the knockout of IL-1R1 either in brain endothelial cells or neurons can offer therapeutic benefits in mouse models of ischemic stroke [21]. Particularly, the genetic deletion of IL-1R1 in the kidneys of autosomal dominant polycystic kidney disease mouse increased the mRNA levels of MLKL, with consequent activation of necroptosis and ferroptosis [22]. In contrast, IL-1R1 was shown to directly interact with RIP1, RIP3, and MLKL, inducing the IL-1R1-necrosome complex formation. As a result, IL-1R1 triggers hemin-induced neuronal necroptosis in mice [23]. Intriguingly, IL-1R antagonist (IL-1RA) can prevent hemin-induced neuronal necroptosis by inhibiting necrosome and maintaining cell membrane integrity in primary hippocampus neurons from mice [23]. Despite these advances, there is no definite evidence of direct association between IL-1R1 and MLKL-dependent necroptosis. Therefore, we hypothesize that hypoxic preconditioning may decrease the expression of IL-1R1 and inhibit the interaction of IL-1R1 with necrosome and ultimately attenuate tGCI-induced neuronal necroptosis via a MLKL-dependent mechanism.

## Materials and methods

In this study, all surgical procedures and animal experiments were performed according to the *Animal Research*: Reporting In Vivo Experiments guidelines and were approved and monitored by the Animal Care and Use Committee of Guangzhou Medical University. Adult male Wistar rats were used for experiments weighting 220 to 280 g (7–8 weeks; Southern Medical University, Guangdong, China). Rats were housed in a temperature-controlled (21–23 °C) and 12-h light/dark cycle environment with free access to food and water. All efforts had been made to minimize both the number of animals used and the suffering of the animals. All animals went through randomization using Random Number Table were divided into different groups according to the standard procedures.

In total, 532 rats were used. Twelve rats died during the tGCI procedure, 9 rats in the tGCI and 4 in the hypoxic preconditioning groups died after tGCI. In addition, 8 rats died after intrahippocampal injection of siRNA, 6 rats died after injection of IL-1RA, 4 died after the lentiviral vector injection, and 13 died during anesthesia. Seventeen rats were excluded due to incomplete occlusion of common carotid arteries and 7 rats that convulsed during ischemia and 2 during 72 h post-ischemia were also excluded.

### Transient global cerebral ischemia and hypoxic preconditioning

The four-vessel occlusion method was used to induce tGCI [24]. Briefly, rats were placed in the anesthesia induction box supplied with 3–4% isoflurane at 3 L/min in 100% oxygen. Anesthesia was maintained with 2–3% isoflurane at 800 ml/min in 100% oxygen and delivered through a nose mask. Vertebral arteries were electrocauterized, and common carotid arteries were isolated and assembled with a teflon/silastic occluding device without blocking blood flow. Global cerebral ischemia was induced in 12-h fasted rats that were awake at 24 h after surgery by occluding both common carotid arteries for 10 min. After occlusion, rats with mydriasis and loss of the righting reflex within 1 min were selected for later experiments. Rectal temperature was maintained at 37–38 °C throughout the procedure, via a rectal probe coupled to a heating lamp. Animals in sham-operated (Sham) group received the same surgical process, except for the occlusion of the common carotid arteries. All operations were conducted by skilled technicians under aseptic conditions. Rats that convulsed during ischemia or post-ischemia were excluded from this study.

Twenty-four hours before ischemia, rats were exposed to systemic hypoxia for the preconditioning process. They were placed in a hypoxic chamber, through which air containing 8% O_2_ and 92% N_2_ flowed continuously at a temperature of 23–25 °C, and were preconditioned for 30 min [25].

### Immunohistochemistry

The animals were sacrificed at 0, 4, 24, 48, and 168 h after reperfusion with or without hypoxia, respectively, and perfused intracardially with 0.9% saline and 4% paraformaldehyde in phosphate-buffered saline (PBS). The brain tissues were removed quickly and postfixed in 10, 20, and 30% sucrose in the same fixative for cytoprotection. After postfixation, the brains were frozen at −20 °C and sliced into coronal 30-μm-thick sections with cryotome (Leica, Wetzlar, Hessen, Germany). Sections selected from the dorsal hippocampus [between anterior-posterior (AP) 4.8 and 5.8 mm, interaural, or AP 3.3 to 3.4 mm, bregma] were used. Single-labeled immunohistochemistry was detected by the avidin-biotin-peroxidase complex (ABC) method [25]. Briefly, the sections were first soaked with 3% hydrogen peroxide for 30 min, followed by 5% normal serum for 1 h at room temperature, and then incubated overnight at 4 °C with primary antibodies (Supplementary file). Afterwards, the slides were washed with PBS (0.01 M, pH 7.4) for three times and then incubated with biotinylated secondary immunoglobulin G antibody for 2 h at room temperature. After being washed with PBS, the sections were treated with ABC for 30 min at room temperature. The peroxidase reaction was visualized with 0.05% diaminobenzidine and 0.01% hydrogen peroxide. Immunopositive cells, in which the reaction product was present within a clear and regular shaped cytoplasmic or nuclear border, were quantified under a light microscope with ×660 magnification. The number or the optical density of immunoreactive cells in CA1 was calculated for 4 nonrepeated random fields (0.037 mm^2^/field × 4 = 0.148 mm^2^ in total). Data were quantified bilaterally in sections from each brain and assessed blindly. Also, four sections for each animal were evaluated.

Double-fluorescent immunohistochemistry was performed as described previously [26]. It was conducted to demonstrate cell types and the exact position where p-MLKL was expressed. NeuN, glial fibrillary acidic protein (GFAP), and ionized calcium binding adaptor molecule-1 (Iba-1) were used to identify neuronal nuclei, astrocytes, and microglia, respectively. The primary antibodies used in these studies were showed in Supplementary file. After being incubated with IgG antibody, sections were washed with PBS and mounted with mounting medium containing 4′,6-diamidino-2-phenylindole (DAPI, Solarbio, Beijing, China, Cat# S2110). Slides were analyzed with a confocal laser microscope (SP8, Leica Microsystems, Wetzlar, Hessen, Germany).

### Western blotting

Rats were sacrificed at 0, 4, 24, and 48 h after reperfusion with or without hypoxia, respectively. The brain tissue was incised into 2-mm coronal slices using a brain matrix, and the CA1 regions of bilateral hippocampi were quickly divided under the stereomicroscope (Supplementary file: Fig. S1). Proteins of hippocampal CA1 subregion were extracted and Western blotting procedure was performed as previously described [25]. In order to detect the compartmental expression of p-MLKL, membranous and cytosolic proteins were extracted with a membrane and cytosol protein extraction kit (Beyotime, Jiangsu, China). To determine protein concentration, bicinchoninic acid (BCA) method was recommended by the manufacturer (Beyotime). The proteins of each sample were separated by sodium dodecyl sulfate-polyacrylamide gel electrophoresis (SDS-PAGE) and then transferred to polyvinylidene fluoride (PVDF) membranes (MilliporeSigma, Burlington, MA, USA). The primary antibodies used in these studies were showed in Supplementary file. Densitometric analysis for the quantification of bands was performed with image analysis software (Quantity One; Bio-Rad, Hercules, CA, USA). Relative optical densities of protein bands were calibrated with GAPDH or Na^+^/K^+^-ATPase and normalized to those in Sham rats.

### Assessment of cellular damage

Animals were sacrificed at 7 days after reperfusion with or without hypoxia and perfused intracardially with 0.9% saline and 4% paraformaldehyde in PBS. The brain tissues were removed quickly and postfixed in 10, 20, and 30% sucrose in the same fixative for cytoprotection. After postfixation, the brains were frozen at −20 °C and sliced into coronal 30-μm-thick sections with cryotome. Sections selected from the dorsal hippocampus were used. As studied previously [27], Nissl, Fluoro- Jade B (F-JB), and NeuN staining were performed to determine the hippocampal cell damage.

The sections from Nissl and NeuN staining were examined under a light microscope (×660). FJ-B stained images were observed with a fluorescent microscope (Leica Microsystems). Cell counts were conducted as described previously [28]. Cells in the CA1 pyramidal layer were quantitatively analyzed within three non-repeated rectangular areas of 0.037 mm^2^. Data were quantified bilaterally in sections from each brain and assessed blindly. Also, four sections for each animal were evaluated.

### Intracellular calcium measurements

Acute brain slices (300 μm) were obtained using a vibratome (Leica VT1200). The slices were then incubated in the dark for 40 min at room temperature in artificial cerebrospinal fluid (aCSF) with 95% O_2_/5% CO_2_. Subsequently, slices were incubated in the dark for 45 min at 37 °C in aCSF containing calcium indicator Fluo-4 AM (5 μmol/L; Thermo Fisher Scientific, Waltham, MA, USA, Cat# F14201) with 95% O_2_/5% CO_2_. After being washed with aCSF for three times, Fluo-4 AM was excited at 488 nm. Stacks of images were rapidly acquired with a confocal laser microscope (SP8, Leica Microsystems). The Ca^2+^ signal was counted within 9 non-repeated rectangular areas (0.012 mm^2^/field × 9 = 0.108 mm^2^ in total) in each CA1. Data were quantified bilaterally in sections from each brain and assessed blindly. Also, 2 sections for each animal were evaluated. Images were analyzed with ImageJ software.

### Immunoprecipitation

Immunoprecipitation procedure was performed as previously described [6]. An amount of 0.6 mg of protein was incubated with rabbit monoclonal antibody against p-MLKL (diluted 1:50; Abcam, Cambridge, MA, USA, Cat# ab196436, RRID: AB_2687465) overnight at 4 °C. The next day, the protein/antibody complex was added to packed protein G agarose beads (MilliporeSigma, Cat# IP05). Following 4 h of incubation at 4 °C, the complex was washed five times with PBS containing 1% Tween. Immunocomplexes were collected by centrifugation and eluted by boiling in loading buffer. The eluted protein samples were subjected to Western blotting with various antibodies (Supplementary file). Densitometric analysis for the quantification of the relative precipitated proteins bands was calibrated with the bands of p-MLKL (ratio of bound to p-MLKL) and normalized to those in Sham rats.

### MLKL siRNA or drug administration

The sequence of MLKL siRNA (GCTACTGTGGGCAGTGATA) was designed by RiboBio (Guangzhou, China) and dissolved with normal saline. As shown in the Supplementary file (Fig. S2), a solution containing 10 μl of MLKL siRNA (2 μmol/L) or the vehicle (normal saline) was administered bilaterally into the hippocampal CA1 region (3.5 mm posterior to bregma, 2.3 mm lateral to bregma, and 2.6 mm below the dura) of rats at 24 h before ischemia. For Sham rats, siRNA or normal saline was administrated at 24 h after operation. The stereotaxic injection was performed over a 10-min period each time using a 10 μl Hamilton syringe with 34-gauge needle at a flow rate of 0.3 μl/min.

A total of 10 μl volume of IL-1RA (0.15 mmol/L, Sino Biological, Beijing, China, Cat# 80073-R01H) or the vehicle (sterile water) were administered bilaterally into the hippocampal CA1 region of rats at 24 h before ischemia.

### Lentivirus construction and lentiviral administration

Plasmids containing the sequence of rat *MLKL* (GenBank accession number XM_008772570) and a negative control sequence (CON319) were designed by Genechem (Shanghai, China). The sequence was inserted into AgeI and AgeI sites of the Ubi-MCS-3FLAG-CMV-EGFP (GV365) lentiviral vector. The shuttle vector and viral packaging system were cotransfected into HEK293T cells to produce recombinant lentiviruses using Lipofectamine 2000 (Invitrogen). Then, HEK293T cells were used for viral infection. The infection efficiency was greater than 80%, as monitored with GFP protein expression. After 48 h of infection with lenti-*MLKL*, the cells were harvested, and total protein was extracted to examine the expression of FLAG. The titers were approximately 0.7 × 10^8^ TU/ml.

Lentiviral administration was carried out as described previously [2]. Briefly, a total of 14 μl volume (10 μl virus diluted by 4 μl enhanced solution) containing 0.7 × 10^6^ TU/ml of particles was injected into bilateral hippocampal CA1 region. The rats were arranged to recover for up to 14 days to enable sufficient gene expression.

### Statistical analysis

Statistical analysis was performed with the Statistical Package for Social Sciences Software for Windows, version 13.0 (SPSS, Inc., Chicago, IL, USA). All variables were expressed as mean ± standard deviation (SD). Statistical significance was determined by one-way ANOVA or two-way analysis followed by a Bonferroni or Tamhane’s T2 post hoc test. The differences were considered statistically significant when *p* < 0.05.

## Results

### Hypoxic preconditioning alleviates tGCI-induced neuronal damage by preventing the upregulation of p-MLKL in CA1

**Fig. 1:**
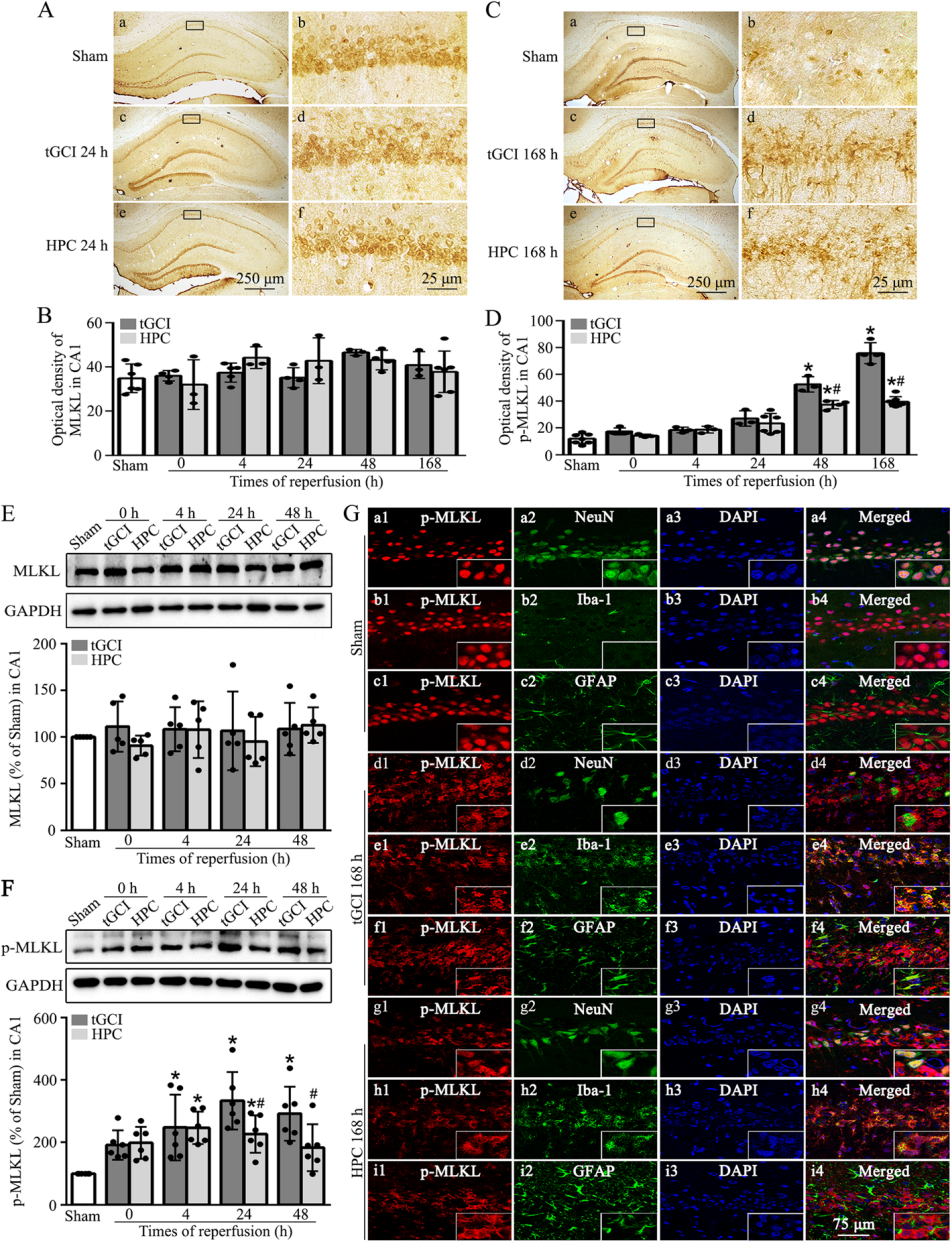
Hypoxic preconditioning prevents the tGCI-induced upregulation of phosphorylated MLKL in CA1. **A** Immunohistochemistry of MLKL in rat brains. Representative images show Sham group (a, b), 24 h after reperfusion of tGCI groups (c, d) and HPC groups (e, f), respectively. **B** Quantitative analyses of MLKL-immunoreactivities in CA1. **C** Immunohistochemistry of p-MLKL (Ser345) in the rat brains. Representative images show Sham group (a, b), 168 h after reperfusion of tGCI groups (c, d) and HPC groups (e, f), respectively. **D** Quantitative analyses of p-MLKL-immunoreactivities in CA1. **E**, **F** Representative immunoblots showing the expression of MLKL and p-MLKL (Ser345) in CA1, respectively. The histogram presents the quantitative analyses of MLKL and p-MLKL (Ser345) levels. Data are expressed as percentage of value of Sham animals. Each bar represents the mean±S.D. ^*^p<0.05 vs. Sham animals and ^#^*p*<0.05 vs. tGCI groups at the same time point. **G** Representative photomicrographs with fluorescent staining of p-MLKL (Ser345) (red), NeuN/Iba-1/GFAP (green) and DAPI (blue) in CA1. HPC hypoxic preconditioning

We first examined the expression of MLKL and p-MLKL by immunohistochemistry and Western blotting. As shown in Fig. 1A, B, MLKL-positive labeling mainly existed in neuron-like cells of pyramidal layer, and no significant increase of MLKL-immunoreactivities was observed in CA1 of tGCI and hypoxic preconditioning rats. Interestingly, compared with Sham group, p-MLKL-immunoreactivities were largely increased in CA1 of tGCI rats at 48 and 168 h after reperfusion. In contrast, the augmentation of p-MLKL-immunoreactivities at 48 and 168 h after reperfusion of tGCI was repressed by hypoxic preconditioning (Fig. 1C, D). Consistent with the results obtained by immunohistochemistry analysis, Western blotting showed no effects of hypoxic preconditioning or tGCI on the expression of MLKL in CA1 (Fig. 1E). Furthermore, p-MLKL was significantly upregulated at 4 h postischemia, and this upregulation persisted through 48 h in CA1 after tGCI, whereas hypoxic preconditioning decreased p-MLKL levels in CA1 after tGCI (Fig. 1F). To determine the cell types that express p-MLKL in CA1 after tGCI with or without hypoxia, we examined cellular localization of p-MLKL by immunofluorescence. As shown in Fig. 1G, almost all p-MLKL-positive cells in Sham rats were NeuN-positive, indicating that p-MLKL was predominantly localized in neurons. Notably, at 168 h after tGCI, most of p-MLKL-positive cells colocalized with Iba-1 but not with GFAP in CA1, indicating microglial localization of p-MLKL. Alternatively, a majority of p-MLKL-positive cells in hypoxic preconditioning rats were both NeuN-positive and Iba-1-positive.

**Fig. 2:**
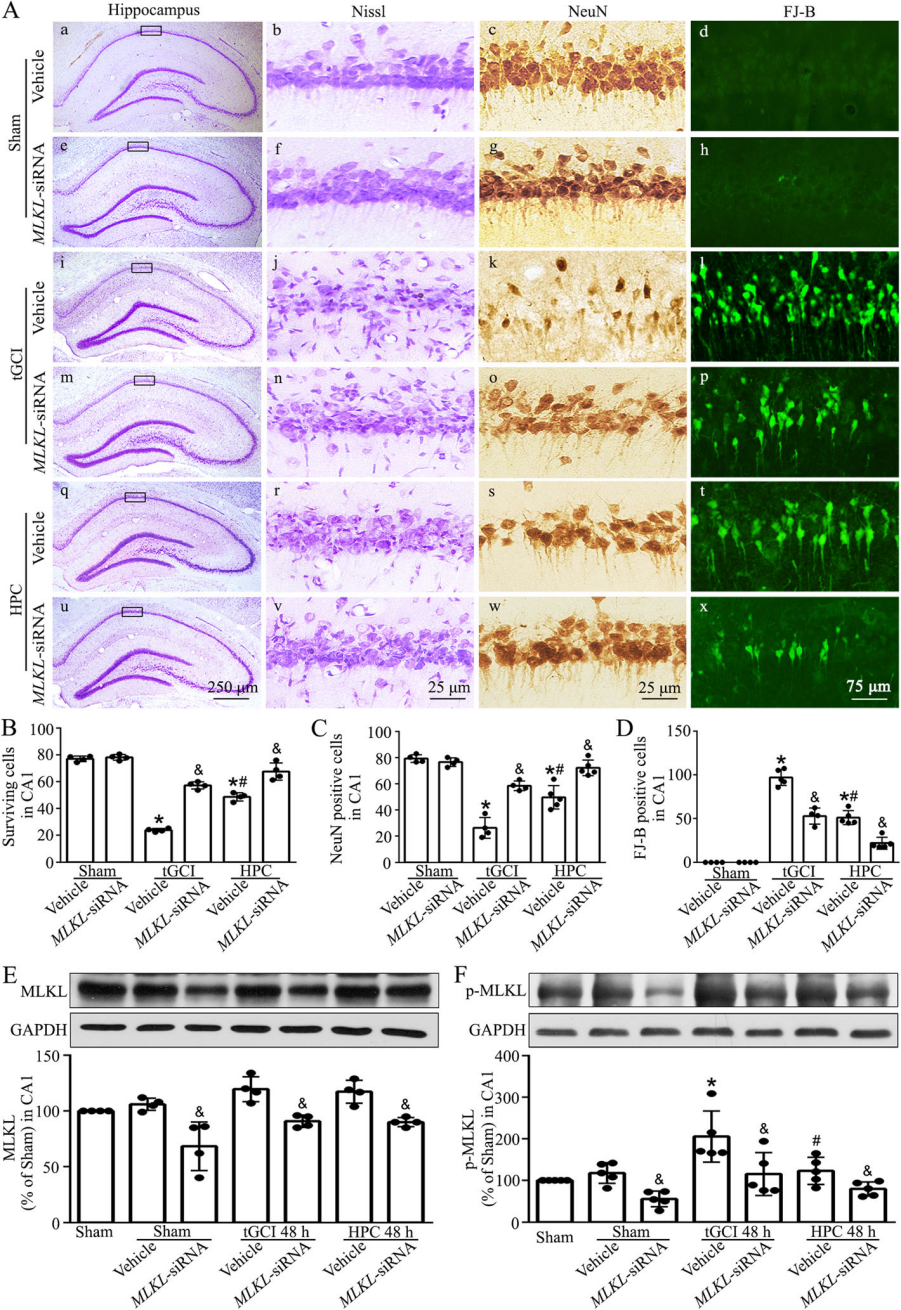
Silencing of MLKL inhibits the phosphorylation of MLKL and alleviates tGCI-induced neuronal damage in CA1. **A** Representative microphotographs of cresyl violet staining, immunostaining of NeuN and FJ-B staining in the hippocampus at 7 days after tGCI with or without MLKL siRNA administration. Sham+Vehicle group, infusion with normal saline (a–d); Sham+MLKL-siRNA group, infusion with MLKL-siRNA (e–h); tGCI+Vehicle group, infusion with normal saline at 24 h before tGCI (i–l); tGCI+MLKL-siRNA group, infusion with MLKL-siRNA at 24 h before tGCI (m–p); HPC+Vehicle group, infusion with normal saline at 24 h before tGCI with hypoxia (q–t); HPC+MLKL-siRNA group, infusion with MLKL-siRNA at 24 h before tGCI with hypoxia (u–x). **B–D** Quantitative analyses of surviving cells, NeuN and FJ-B-positive cells in CA1. Each bar represents the mean±S.D. ^*^p<0.05 vs. Sham+Vehicle animals, ^#^*p*<0.05 vs. tGCI+Vehicle group and ^&^*p*<0.05 vs. tGCI or HPC group administrated with vehicle. **E**, **F** Representative images of western blotting showing the expression of MLKL and p-MLKL (Ser345) in CA1 after tGCI with or without MLKL-siRNA administration. The histogram presents the quantitative analyses of MLKL and p-MLKL (Ser345) in CA1. Data are expressed as percentage of value of Sham animals. Each bar represents the mean±S.D. ^*^p<0.05 vs. Sham animals, ^#^*p*<0.05 vs. tGCI+Vehicle group and ^&^*p*<0.05 vs. Sham or tGCI or HPC group administrated with vehicle. HPC hypoxic preconditioning

To validate the causal role of MLKL-related neuronal necroptosis induced by tGCI, siRNA-mediated knockdown of MLKL was used at 24 h before tGCI. As shown in Fig. 2A–D, compared with Sham rats treated with vehicle, the neuronal damage was extremely aggravated in CA1 at 7 days after tGCI, accompanied by a decrease in surviving and NeuN-positive cells and an increase in FJ-B-positive cells. Noteworthily, hypoxic preconditioning or MLKL knockdown alleviated aforementioned neuronal damage. Additionally, a cumulative neuroprotective effect was observed when hypoxic preconditioning and MLKL siRNA were combined. Accordingly, MLKL siRNA treatment significantly inhibited the expression of MLKL and the phosphorylation of MLKL in CA1 at 48 h after tGCI (Fig. 2E, F).

**Fig. 3:**
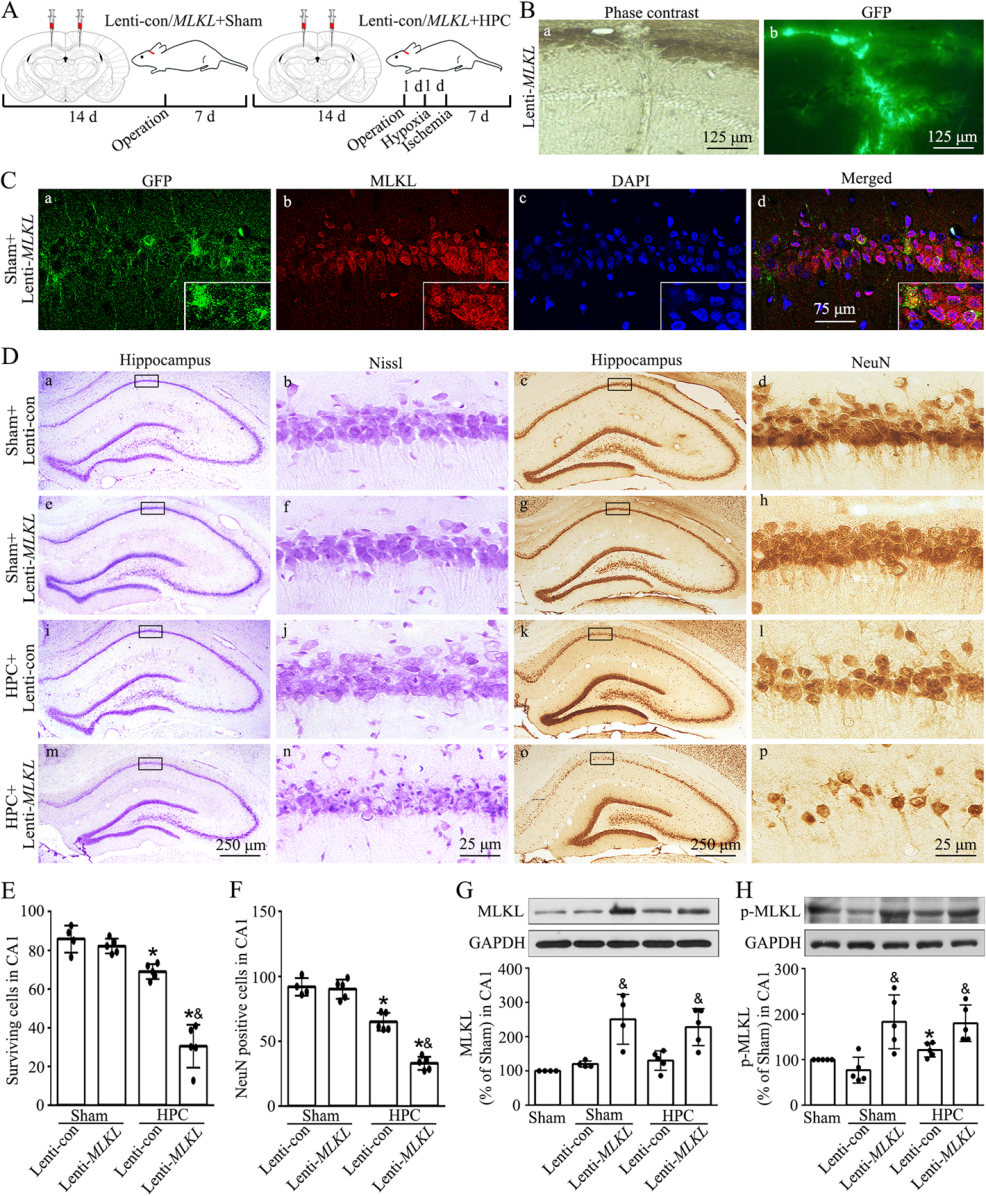
Overexpression of MLKL increases phosphorylated MLKL and reverses the hypoxic preconditioning-induced neuroprotection in CA1 after tGCI. **A** Design of experiments in which rats were stereotaxically injected bilaterally with MLKL lentiviral vectors in the dorsal CA1 pyramidal layer and subjected to either Sham or tGCI with hypoxia. **B** Phase contrast and fluorescent images from coronal sections of CA1 following injection of MLKL lentiviral vectors in Sham animals. **C** Representative photomicrographs show the co-localization of GFP (green), MLKL (red), and DAPI (blue) in CA1 from Sham animals with Lenti-*MLKL* injection. **D** Cresyl violet stained and NeuN immunostained hippocampal sections from rats administered bilaterally with either Lenti-control or Lenti-*MLKL* at 7 days after reperfusion of tGCI with hypoxia. Boxes indicate that the magnified regions displayed in the right panel. **E**, **F** Quantitative analyses of surviving cells and NeuN-positive cells in CA1. Each bar represents the mean±S.D. ^*^p<0.05 vs. Sham+Lenti-con animals, and ^&^*p*<0.05 vs. HPC group with Lenti-con. **G**, **H** Representative immunoblots of MLKL and p-MLKL (Ser345) expression in CA1 after hypoxic preconditioning with or without MLKL lentiviral vector administration. The histogram presents the quantitative analyses of MLKL or p-MLKL protein. Data are expressed as percentage of value of Sham animals. Each bar represents the mean±S.D. ^*^p<0.05 vs. Sham animals, and ^&^*p*<0.05 vs. Sham or HPC group with Lenti-con. Lenti-con, Lenti-Conrtol, scrambled lentivirus vector; Lenti-*MLKL MLKL*-carried lentivirus; HPC hypoxic preconditioning

Further, to confirm whether hypoxic preconditioning specifically abrogates the MLKL-related neuronal necroptosis, lentivirus vectors mediated MLKL overexpression (LV-*MLKL*), or the negative control vectors (LV-control) were injected into the bilateral hippocampal CA1 at 14 days before the 4-vessel occlusion surgery (Fig. 3A, Supplementary file: Fig. S3A). As shown by immunofluorescence, MLKL was colocalized with GFP in LV-*MLKL* administrated rats at 7 days after surgery operation (Fig. 3C). As expected, the administration of LV-*MLKL* had no neurotoxic effects on the neurons in CA1 of Sham rats. Also, the overexpression of MLKL via LV-*MLKL* administration did not aggravate the neuronal damage in CA1 of tGCI rats (Supplementary file: Fig. S3B-D). However, it eliminated the protective roles of hypoxic preconditioning in ischemic neuronal damage in CA1 (Fig. 3D–F). Accordingly, the expressions of MLKL and p-MLKL in CA1 detected by Western blotting increased after the administration of LV-*MLKL* either in Sham or tGCI with or without hypoxia rats (Fig. 3G, H; Supplementary file: Fig. S3E, F).

### Hypoxic preconditioning blocks the membranous translocation of p-MLKL and the influx of calcium ions in CA1 after tGCI

**Fig. 4:**
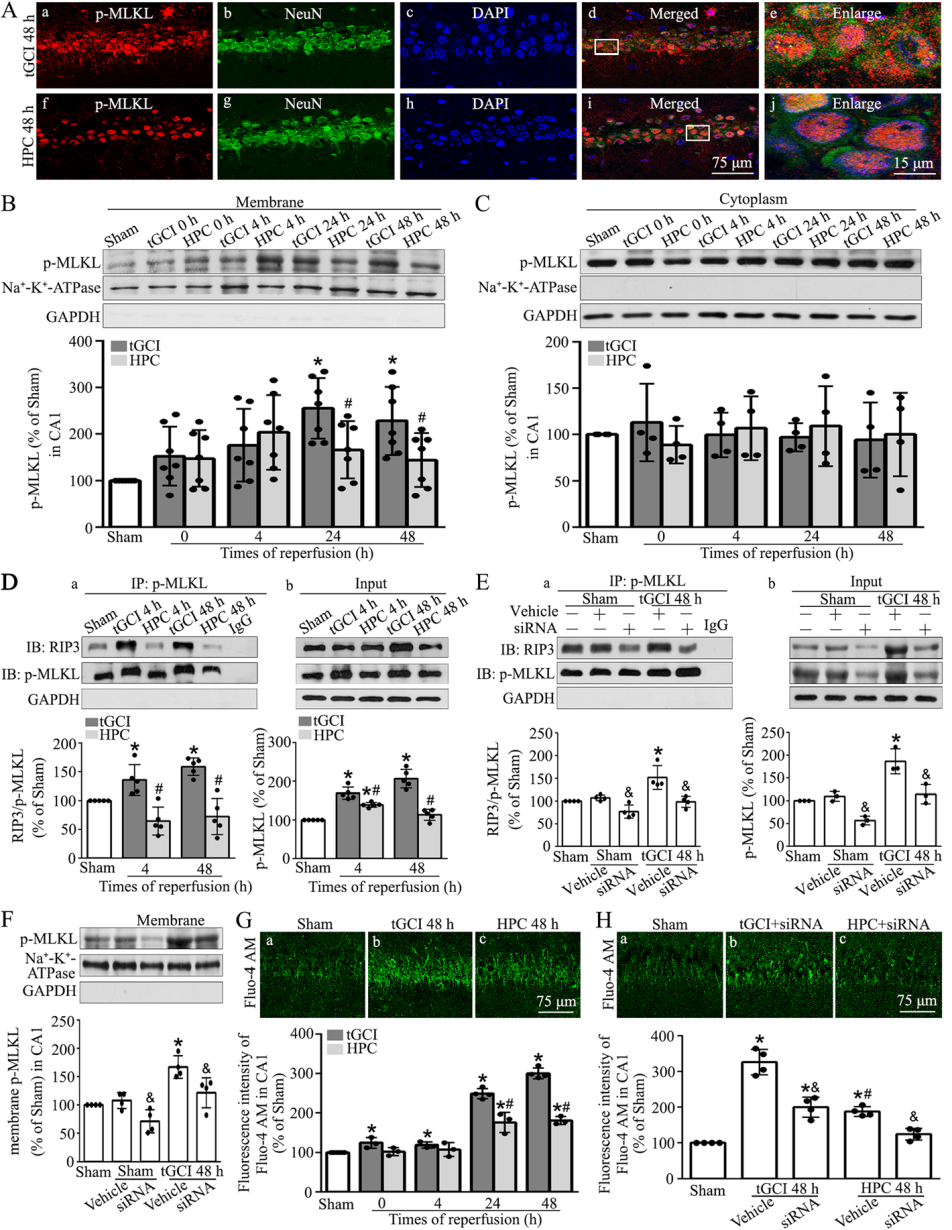
Hypoxic preconditioning blocks the tGCI-induced plasma membrane translocation of p-MLKL and calcium influx in CA1. **A** Representative photomicrographs with fluorescent staining of p-MLKL (red), NeuN (green), and DAPI (blue). **B**, **C** Representative immunoblots and quantitative data show p-MLKL in plasma membrane and cytosolic proteins, respectively. **D** Immunoprecipitation and western blot assays showing the effects of hypoxic preconditioning on the formation of RIP3-p-MLKL **(D, a)** and the expression of RIP3 and p-MLKL **(D, b)** after tGCI. Quantification of the relative precipitated proteins is expressed as a ratio of precipitates to input. **E** Immunoprecipitation and western blot assays showing the effects of MLKL siRNA on the formation of RIP3-p-MLKL **(E**, **a)** and the expression of RIP3 and p-MLKL **(E**, **b)** after tGCI. **F** Effects of pretreatment with MLKL siRNA on the p-MLKL expression in plasma membrane protein in CA1 of tGCI rats using immunoblot analysis. **G** Representative intracellular calcium concentration in CA1 after tGCI with or without hypoxia. Representative images show Sham group (a), 48 h after reperfusion of tGCI group (b) and hypoxic preconditioning group (c), respectively. Quantitative analysis of fluorescence intensity of Fluo-4-AM in CA1. **H** Representative intracellular calcium concentration in CA1 after tGCI with or without MLKL siRNA administration. Sham group (a), tGCI+MLKL-siRNA group, infusion with MLKL-siRNA at 24 h before tGCI (b), HPC+MLKL-siRNA group, infusion with MLKL-siRNA at 24 h before tGCI with hypoxia (c). Data are expressed as percentage of value of Sham animals. Each bar represents the mean±S.D. ^*^p<0.05 vs. Sham animals, ^#^*p*<0.05 vs. tGCI groups at the same time point, and ^&^*p*<0.05 vs. tGCI or hypoxic preconditioning group with injection of MLKL siRNA. IP immunoprecipitation, IB immunoblotting, HPC hypoxic preconditioning

To better define the molecular mechanism by which p-MLKL acts, we analyzed p-MLKL expression in the membrane and non-membrane fractions. As shown in Fig. 4A, p-MLKL signals in CA1 at 48 h after tGCI were only partially detected surrounding NeuN signals, signifying the distribution of p-MLKL in the plasma membrane after tGCI. Compared to the tGCI rats, p-MLKL signals overlapped with both NeuN and DAPI signals in CA1 at 48 h of hypoxic preconditioning rats, suggesting that hypoxic preconditioning attenuates the translocation of p-MLKL to the plasma membrane in CA1 after tGCI. To further quantify the expression of p-MLKL in cellular compartments, we also extracted membrane proteins from CA1 of tGCI rats and hypoxic preconditioning rats. Consistently, as shown in Fig. 4B, membrane-associated p-MLKL levels increased at 24 and 48 h after tGCI, and they were obviously reduced with hypoxic treatment before tGCI. However, no significant differences were observed in cytosolic p-MLKL levels between rats in tGCI and hypoxic preconditioning groups (Fig. 4C). Nevertheless, the increase of p-MLKL levels in membranous fraction induced by tGCI was diminished due to the treatment with MLKL siRNA (Fig. 4F).

Findings on RIP3-MLKL interaction in mediating necroptosis through MLKL membranous translocation [29] prompted our investigation on the interaction of RIP3 and p-MLKL by immunoprecipitation. An obvious increase in the RIP3-p-MLKL interaction was observed in tGCI group at 4 and 48 h after reperfusion (Fig. 4D). On the other hand, hypoxic preconditioning significantly attenuated the RIP3-p-MLKL interaction. With MLKL siRNA treatment the result was similar to that of hypoxic pretreatment at 48 h after tGCI (Fig. 4E).

Due to the fact that Ca^2+^ influx into cells act as a downstream factor of MLKL during necroptosis [11], we hereafter examined whether Ca^2+^ influx was involved in tGCI-induced neuronal necroptosis. Intracellular free calcium concentration was measured using Fluo-4 AM fluorescence indicator. Marked increases in Fluo-4 AM fluorescence intensity of CA1 were shown at 24 and 48 h after tGCI. In contrast, the treatment either with hypoxic preconditioning or MLKL silencing attenuated tGCI-induced Ca^2+^ influx in CA1 (Fig. 4G, H).

### Hypoxic preconditioning alleviates MLKL-mediated neuronal necroptosis via downregulating IL-1R1 in CA1 after tGCI

**Fig. 5:**
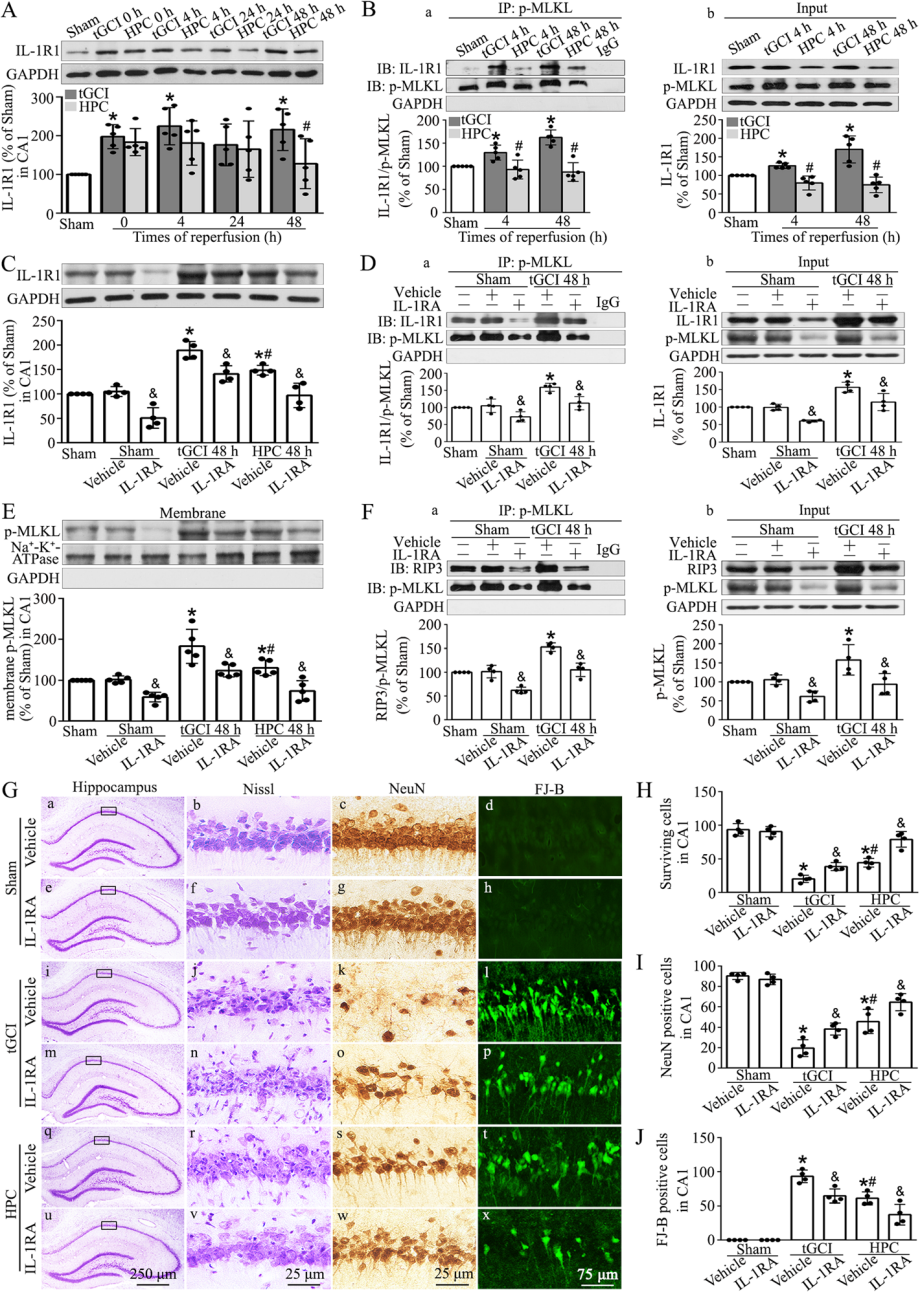
Hypoxic preconditioning downregulates the expression of IL-1R1 and alleviates p-MLKL-dependent neuronal damage in CA1 after tGCI. **A** Western blot analysis of IL-1R1 in CA1. The histogram presents the quantitative analyses of IL-1R1 levels. **B** Immunoprecipitation and western blot assays showing the effects of hypoxic preconditioning on the formation of IL-1R1-p-MLKL **(B, a)** and the expression of IL-1R1 and p-MLKL **(B, b)** in CA1 of tGCI. **C** Representative images of western blotting showing the expression of IL-1R1 in CA1 after tGCI with or without IL-1RA administration. **D** Immunoprecipitation and western blot assays showing the effects of IL-1RA on the formation of IL-1R1-p-MLKL **(D, a)** and the expression of IL-1R1 and p-MLKL **(D, b)** in CA1 of tGCI. **E** Effects of pretreatment with IL-1RA on the p-MLKL expression in plasma membrane protein in CA1 of tGCI rats with or without hypoxia using immunoblot analysis. **F** Immunoprecipitation and western blot assays showing the effects of IL-1RA on the formation of RIP3-p-MLKL **(F, a)** and the expression of RIP3 and p-MLKL **(F, b)** in CA1 of tGCI. **G** Representative microphotographs of cresyl violet staining, immunostaining of NeuN and FJ-B staining in the hippocampus at 7 days after tGCI with or without IL-1RA administration. **H–J** Quantitative analyses of surviving cells, NeuN and FJ-B-positive cells in CA1. Each bar represents the mean±S.D. ^*^p<0.05 vs. Sham+Vehicle animals, ^#^*p*<0.05 vs. tGCI+Vehicle group and ^&^*p*<0.05 vs. tGCI or HPC group administrated with vehicle. IP immunoprecipitation, IB immunoblotting, HPC hypoxic preconditioning

It is known that IL-1R1 interacts with RIP1, RIP3, and MLKL, thereby inducing the formation of IL-1R1-necrosome complex and triggering neuronal necroptosis [23]. We thus investigated the expression of IL-1R1. As shown in Fig. 5A, IL-1R1 expression in CA1 was largely upregulated at 0, 4, and 48 h after reperfusion by tGCI injury, but restored to basic level by hypoxic pretreatment. Additionally, with immunoprecipitation, we found an enhanced interaction of IL-1R1 and p-MLKL in tGCI group at 4 and 48 h after reperfusion. On the contrary, when rats were subjected to hypoxic preconditioning, the interaction of IL-1R1 and p-MLKL was attenuated (Fig. 5B).

When IL-1RA, an antagonist of IL-1R1, was used, IL-1R1 expression in CA1 was inhibited at 48 h after tGCI (Fig. 5C). However, neutralizing IL-1R1 with IL-1RA did not alter the expression of MLKL after tGCI (Supplementary file: Fig. S4). As expected, the expression of p-MLKL and the translocation of p-MLKL to the plasma membrane were attenuated (Fig. 5Db, E), and the interactions of IL-1R1-p-MLKL and RIP3-p-MLKL were interrupted (Fig. 5D, F). In addition, the inhibition of IL-1R1 with IL-1RA treatment significantly alleviated neuronal death in CA1 at 168 h after tGCI (Fig. 5G–J).

## Discussion

**Fig. 6:**
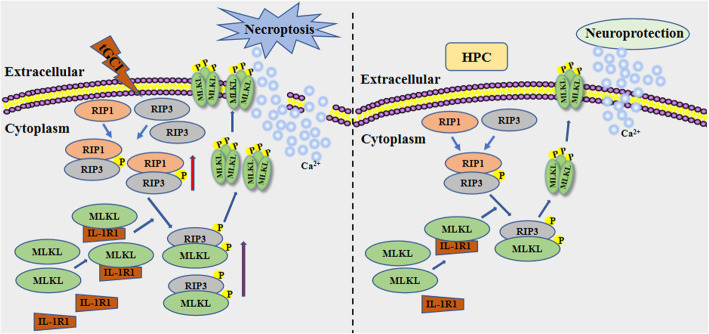
Schematic depicting mechanism by which hypoxic preconditioning protects neuron against tGCI in the hippocampal CA1 through inhibition of MLKL-dependent necroptosis. The expression of RIP3 and the interaction of RIP1-RIP3 were increased in CA1 after reperfusion of tGCI. In addition, tGCI enhanced the expression of IL-1R1, which in turn, increased the RIP3-p-MLKL interaction, promoted the plasma membrane translocation of p-MLKL and the influx of Ca^2+^, ultimately inducing neuronal necroptosis. Hypoxic preconditioning downregulated the expression of IL-1R1, disrupted the interaction between IL-1R1 and the necrosome, attenuated the expression and the plasma membrane translocation of p-MLKL, thus decreasing the Ca^2+^ influx and alleviating neuronal death in CA1 after tGCI. HPC hypoxic preconditioning

In the current study, we demonstrates that hypoxic preconditioning downregulates the phosphorylation of MLKL in CA1 after tGCI. The knockdown of MLKL with siRNA in CA1 after tGCI effectively reduces MLKL and p-MLKL expression and attenuates neuronal death in CA1 after cerebral ischemia, whereas overexpression of MLKL with lentiviral delivery of MLKL showed opposite results. Mechanistically, pretreatment with hypoxic preconditioning or MLKL siRNA inhibits the RIP3-p-MLKL interaction, disrupts the plasma membranous translocation of p-MLKL, and attenuates the Ca^2+^ influx in CA1 after tGCI. In addition, hypoxic preconditioning blocks the necrosome complex formation with IL-1R1. Disrupting the interaction between IL-1R1 and the necrosome by injecting IL-1RA can inhibit the activation of MLKL and protect neurons from tGCI-induced cell death (Fig. 6). This is by far the first study to reveal the involvement of IL-1R1 in tGCI-induced neuronal necroptosis mediated by a MLKL-dependent mechanism.

Necroptosis, a programmed form of necrosis, which is distinct from apoptosis and necrosis, involves in a range of human diseases including cerebral ischemic injury as a new mechanism of cell death [6, 30]. We had recently reported that necroptosis contributed to tGCI-induced pyramidal neuronal death in CA1, and hypoxic preconditioning alleviated necroptotic neuronal death after tGCI [6]. The signaling pathway responsible for carrying out necroptosis after cerebral ischemia has not been fully understood. It has been shown that the phosphorylation of MLKL on its kinase domain by RIP3 is essential for MLKL to function in necroptosis [13]. As MLKL is a pseudokinase, it cannot conduct necrosis signals through phosphorylation of downstream effectors. Hence, MLKL is considered to be a terminal executor of necroptosis. To explore whether MLKL was involved in neuronal necroptosis induced by tGCI, we detected the expression of MLKL. Here, we demonstrate that hypoxic preconditioning downregulated p-MLKL expression and attenuated RIP3 and p-MLKL interaction without affecting MLKL expression in CA1 after tGCI. Moreover, using siRNA of MLKL to decrease the expression of MLKL, the phosphorylation of MLKL and the interaction between RIP3 and p-MLKL not only was suppressed, but also neuronal damage after tGCI was alleviated. Also, the overexpression of MLKL abrogated the neuroprotection of hypoxic preconditioning. Based on these observations, we propose that the phosphorylation of MLKL after tGCI is essential in mediating neuronal necroptosis.

It is generally believed that the types of cells that undergo necroptosis may be switched after cerebral ischemia. Recent studies indicated necroptosis in neurons at the early stage but in astrocytes at the later stage after focal cortical ischemia in mice [31]. Similarly, by examining the expression of p-MLKL, we observed that a neuron-dominant necroptosis occurred in the first 48 h after tGCI. However, at 168 h after tGCI, the major cell type, which underwent necroptosis, may switch from neuron to microglia. Huang et al. reported that microglia underwent necroptosis and released cytokines and chemokines, thus exacerbating neural damage and degeneration both in the retinal degenerative rd1 mice and in the acute retinal neural injury mice [32]. Therefore, we speculate that this time-dependent switch of MLKL to microglia reflects the pathological changes of cerebral ischemia and can contribute to the development of secondary injury. In addition, it is known that microglia have three phenotypes: ramified (resting), activated and ameboid (macrophagic) microglia, and the latter has the functions similar to those of macrophages, including the ability to engulf 5 micron latex beads, such as debris [33]. Therefore, it is possible that the transition of p-MLKL from neurons to microglia after tGCI reflects the macrophagic role of microglia to injured neurons. Further investigation will be required to elucidate this interesting mechanism.

How does MLKL execute necroptosis? Multiple mechanisms including the integrity of mitochondria and lysosomes, and the generation of reactive oxygen species (ROS) and Ca^2+^ signaling have been proposed [34]. A study of Wang et al. implicates the mitochondrial proteins phosphoglycerate mutase family member 5 (PGAM5) and dynamin-related protein 1 (Drp1) as effectors activated by MLKL [35]. Previously, we demonstrated that the inhibition of Drp1 blocked neuronal necroptosis after tGCI [6], suggesting that MLKL activates Drp1 to mediate neuronal necroptosis. However, in this study, phosphorylated MLKL was mainly distributed in the nucleus of Sham and hypoxic preconditioning rats brain, and it translocated to the plasma membrane after tGCI. These results reveal that phosphorylated MLKL-induced neuronal necroptosis after tGCI may be independent on Drp1-mediated mitochondrial pathway. MLKL has been observed in various subcellular compartments, such as the cytosol [13], mitochondrial fraction [35, 36], and plasma membrane [11, 18, 37, 38]. Notably, phosphorylated MLKL can form oligomers and move from cytoplasm to the cell membrane, resulting in the formation of the pore, causing an inflammatory response [39]. In spite of pore formation, the plasma membrane translocation of MLKL binds to phosphatidylinositol lipids and cardiolipin, and directly disrupts membrane integrity, and finally leads to cell death [14]. Here, we find that phosphorylated MLKL translocates to the plasma membrane after tGCI, which may in turn induce neuronal death.

As mentioned above, activated MLKL causes directly plasma membrane rupture by binding with negative charged lipids of membrane which drives lytic cell death [14]. In contrast, MLKL was proved to form selective channels and cause the intracellular ion dysregulation. For instance, evidence suggests that membrane translocation of MLKL resulted in Ca^2+^ influx [11]. The elevated cytosolic Ca^2+^ increases reactive oxygen species (ROS) and intracellular acidification, decreases adenosine triphosphate, and ultimately leads to plasma membrane rupture [40] and cell excitotoxic injury. Recently, a study of Zhu et al. implicated that Ca^2+^ influx in cardiomyocytes treated with lipopolysaccharide and H_2_O_2_ raised cellular ROS and mediated the mitochondrial permeability transition pore opening, thus promoting cardiomyocytes necroptosis [41]. In this study, we demonstrated that the intracellular Ca^2+^ concentration was markedly increased in CA1 after tGCI and that MLKL silencing or hypoxic preconditioning abolished Ca^2+^ influx in response to tGCI in CA1. Previously, we had reported the gradually increased level of ROS in CA1 over time after the reperfusion of tGCI [42]. These results indicate that Ca^2+^ influx mediated by the activation of MLKL may increase ROS and promote neuronal necroptosis after tGCI. In contrast to our observations, González-Juarbe et al. reported that the dysregulation of ions, including Ca^2+^ influx and K^+^ efflux, is sufficient to trigger the necroptosis machinery, resulting in MLKL activation and mitochondrial damage in respiratory epithelial cells during bacterial pneumonia [43]. Thus, the dysregulation of ions may function as an initiator for the regulatory mechanism of necroptosis. However, it remains to be illuminated whether MLKL-induced killing involves other cellular factors, such as ion channel opening. Future study should address how MLKL regulates Ca^2+^ influx in plasma membrane.

Recently, IL-1R1 has been proposed to interact with MLKL and trigger hemin-induced neuronal necroptosis [23]. Our experiments revealed an immediate and enhanced expression of IL-1R in CA1 after tGCI, indicating that IL-1R1 was involved in neuronal damage induced by tGCI. Furthermore, injection with IL-1RA, an antagonist of IL-1R1, diminished neuronal death in CA1 after tGCI. Similarly, in the rodent models of focal cerebral ischemia or excitotoxic damage induced by NMDA-receptor agonist, with IL-1RA treatment, the neuroprotective effects were substantiated [44, 45]. These studies expand clinical indications of IL-1RA as a neuroprotectant after ischemic stroke. Of note, our study further demonstrated the requirement of IL-1R1 for necrosome formation, providing new evidence of controlling activities of MLKL during the process of neuronal necroptosis. For the first time, a significant interaction between IL-1R1 and p-MLKL in CA1 from rats with tGCI was verified. Furthermore, the inhibition of IL-1R1 decreased the phosphorylation of MLKL and interrupted the interaction of IL-1R1 and p-MLKL after tGCI. Importantly, this inhibition reduced ulteriorly the translocation of p-MLKL to the plasma membrane and alleviated neuronal death. Our results confirm that the interaction between IL-1R1 and p-MLKL is crucial to trigger neuronal necroptosis after tGCI.

## Conclusions

Our study demonstrates that tGCI triggers neuronal necroptosis via the induction of IL-1R1-p-MLKL complex formation. Hypoxic preconditioning blocks the necrosome complex formation with IL-1R1 and inhibits the activation and membranous translocation of MLKL, which in turn prevents Ca^2+^ influx and ultimately protects neurons from tGCI-induced cell necroptosis. Our results solidify the notion that necroptosis occurs following tGCI-induced hippocampal neuronal damage and add to the existent body of evidence suggesting that blocking necroptosis with hypoxic preconditioning can protect against neuronal injury during cerebral ischemia, and that MLKL may be a potential therapeutic target for the treatment of cerebral ischemia.

## Supplementary Information

**Additional file 1.** Supplementary methods and figures.

## Data Availability

The data supporting the conclusions of this article are included within the article. Original slides, photographs, and blot images are retained. All regents used in this study are available from scientific supply companies. The datasets used and analyzed during the current study are available from the corresponding author on reasonable request.

## Abbreviations

Ca^2+^: Calcium ion
Drp1: Dynamin-related protein 1
F-JB: Fluoro-Jade B
GFAP: Glial fibrillary acidic protein
Iba-1: Ionized calcium-binding adaptor molecule-1
IL-1R1: Interleukin-1 type 1 receptor
IL-1RA: IL-1R antagonist
NeuN: Neuronal nuclei
MLKL: Mixed lineage kinase domain-like
p-MLKL: Phosphorylated MLKL
RIP: Receptor interacting protein
tGCI: Transient global cerebral ischemia
