# Supplementary material for: Inhibition of MLKL-dependent necroptosis via downregulating interleukin-1R1 contributes to neuroprotection of hypoxic preconditioning in transient global cerebral ischemic rats
Source: J Neuroinflammation. 2021 Apr 20;18:97. doi: 10.1186/s12974-021-02141-y (PMC8056617; doi:10.1186/s12974-021-02141-y)
Supplement: Supplementary file 1 — Additional file 1. Supplementary methods and figures. [file 12974_2021_2141_MOESM1_ESM.pdf]

## Supplementary

### Supplementary Methods:

**Primary antibodies for single-labelled immunohistochemistry**

Primary antibodies include mouse monoclonal antibody against MLKL (diluted 1:2000; MilliporeSigma, Burlington, MA, USA, Cat# MABC604, RRID: AB_2820284), rabbit monoclonal antibody against phosphorylated MLKL (p-MLKL) (Ser345; diluted 1:2000; Abcam, Cambridge, MA, USA, Cat# ab196436, RRID: AB_2687465), and mouse monoclonal antibody against neuronal nuclei (NeuN; diluted 1:6000; MilliporeSigma, Cat# MAB377, RRID: AB_2298772).

**Primary antibodies for double-fluorescent immunohistochemistry**

Primary antibodies include rabbit monoclonal antibody against p-MLKL (Ser345; diluted 1:200; Abcam, Cat# ab196436, RRID: AB_2687465), mouse monoclonal antibody against NeuN (diluted 1:3000; MilliporeSigma, Cat# MAB377, RRID: AB_2298772), mouse monoclonal antibody against Iba-1 (diluted 1:100; Wako, Chuo-ku, Osaka, Japan, Cat# 016-26721, RRID: AB_2811160), mouse anti-GFAP (diluted 1:3000; MilliporeSigma, Cat# MAB360, RRID: AB_11212597), Cy3-conjugated goat anti-rabbit IgG antibody (diluted 1:100; MilliporeSigma, Cat# AP132C, RRID: AB_92489), and FITC-conjugated goat anti-mouse IgG antibody (diluted 1:100; MilliporeSigma, Cat# AP308F, RRID: AB_92634).

**Primary antibodies for western blotting**

The primary antibodies include mouse polyclonal antibody against MLKL (diluted 1:1000; MilliporeSigma, Cat# MABC604, RRID: AB_2820284), rabbit monoclonal antibody against p-MLKL (Ser345; diluted 1:1000; Abcam, Cat# ab196436, RRID: AB_2687465), rat monoclonal antibody against IL-1R1 (diluted 1:1000; Abcam, Cat# ab8154, RRID: AB_306312), mouse monoclonal antibody against glyceraldehyde 3-phosphate dehydrogenase (GADPH; diluted 1:10,000; Proteintech Group, Chicago, IL, USA, Cat# 60004-I-Ig, RRID: AB_2107436), and rabbit monoclonal antibody against $Na^+/K^+$-ATPase (diluted 1:1000; Cell Signaling Technology, Danvers, MA, USA, Cat# 3010, RRID: AB_ 2060983).

**Primary antibodies for immunoprecipitation**

The primary antibodies include rabbit monoclonal antibody against RIP3 (diluted 1:1000; Prosci, Fort Collins, CO, USA, Cat# 2283, RRID: AB_203256), rat monoclonal antibody against IL-1R1 (diluted 1:1000; Abcam, Cat# ab8154, RRID:

AB_306312), and rabbit monoclonal antibody against p-MLKL (diluted 1:1000; Abcam, Cat# ab196436, RRID: AB_2687465).

**Supplementary Figures**

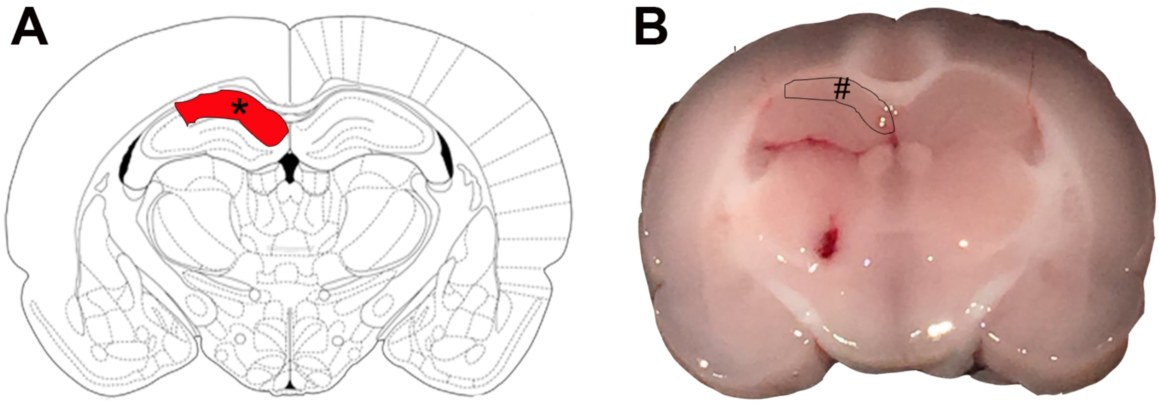


**Fig. S1 Schematic illustration of hippocampal CA1 region. A (*red area) and B (#light brown area) indicates hippocampal CA1 region, which is located within the area from bregma -2.30 mm to -4.52 mm based on the rat brain atlas by Paxinos and Watson (1997).**

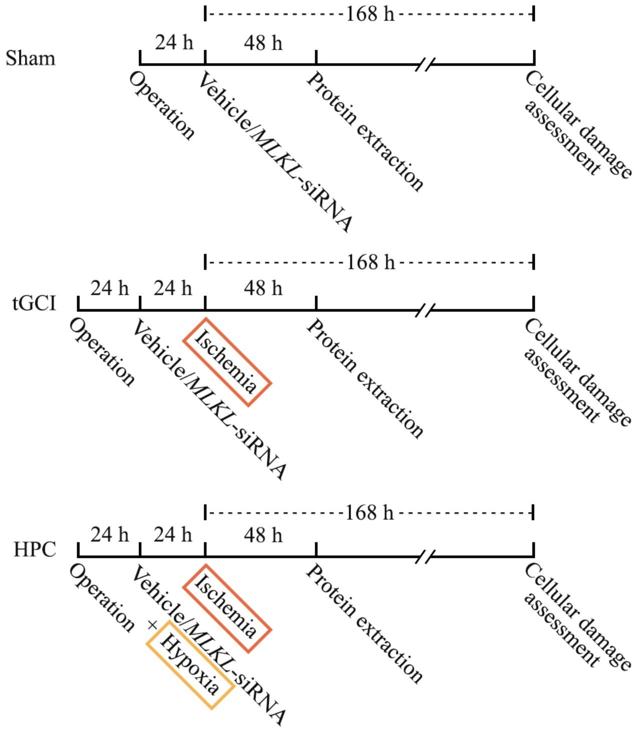


**Fig. S2 Design of experiments in which rats were stereotaxically injected bilaterally with MLKL-siRNA in the dorsal CA1 pyramidal layer and subjected to either Sham or tGCI with or without hypoxia.**

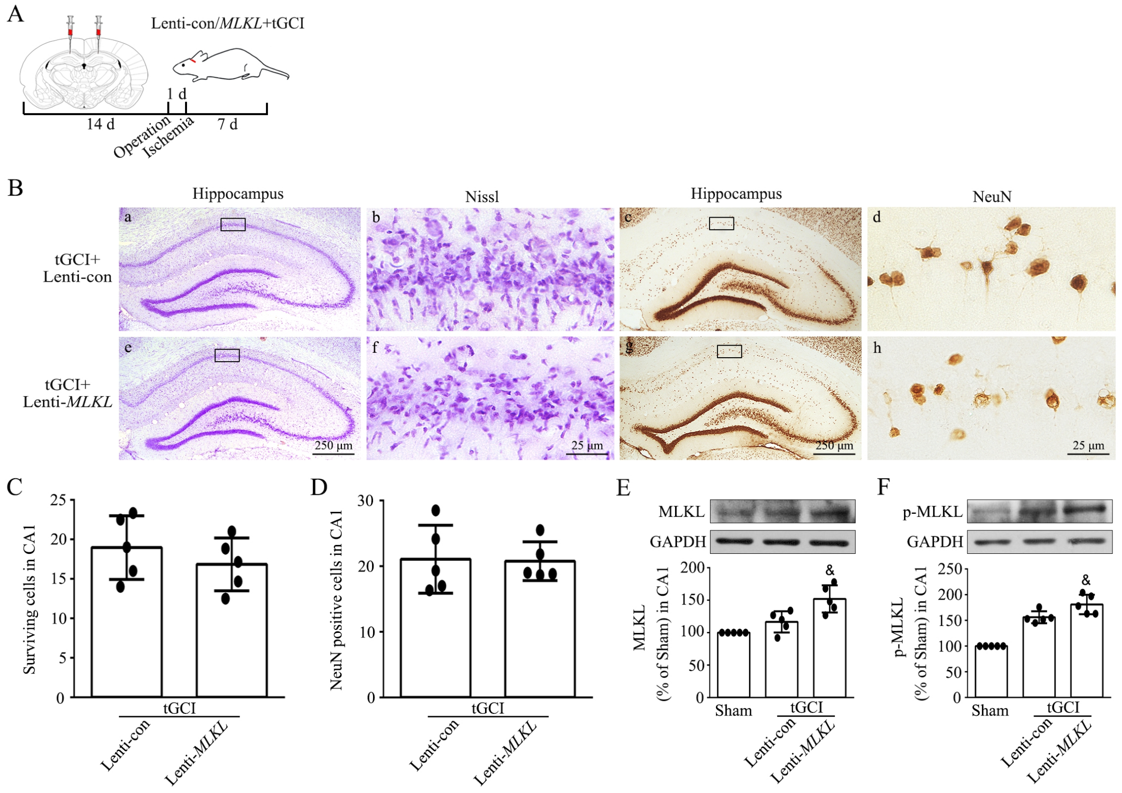


**Fig. S3 Overexpression of MLKL increases phosphorylated MLKL, but not aggravates the neuronal damage in CA1 after tGCI. (A)** Design of experiments in which rats were stereotaxically injected bilaterally with MLKL lentiviral vectors in the dorsal CA1 pyramidal layer and subjected to tGCI. **(B)** Cresyl violet stained and NeuN immunostained hippocampal sections from rats administered bilaterally with either Lenti-control or Lenti-*MLKL* at 7 d after reperfusion of tGCI. Boxes indicate that the magnified regions displayed in the right panel. **(C & D)** Quantitative analyses of surviving cells and NeuN-positive cells in CA1. Each bar represents the mean±S.D. **(E & F)** Representative immunoblots of MLKL and p-MLKL (Ser345) expression in CA1 after tGCI with either Lenti-control or Lenti-*MLKL* administration. The histogram presents the quantitative analyses of MLKL or p-MLKL protein. Data are expressed as percentage of value of Sham animals. Each bar represents the mean±S.D. $^{\&}p<0.05$ vs. tGCI group with Lenti-con. Lenti-con, Lenti-Conrtol, scrambled lentivirus vector; Lenti-*MLKL*, *MLKL*-carried lentivirus.

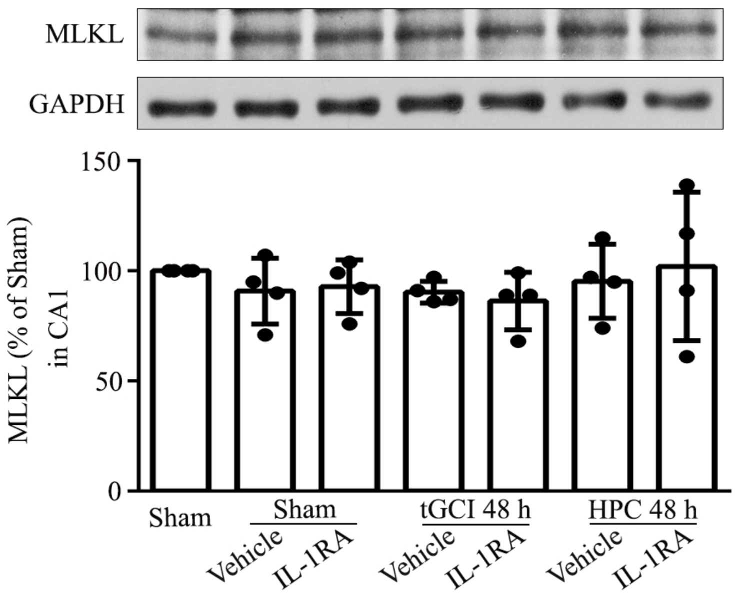


**Fig. S4 Effects of pretreatment with IL-1RA on the MLKL expression in CA1 of tGCI rats with or without hypoxia using immunoblot analysis.** Each bar represents the mean±S.D. HPC, hypoxic preconditioning.
